# ﻿First record of the genus *Camptotheca* (Nyssaceae) in Vietnam and the lectotypification of C.acuminata

**DOI:** 10.3897/phytokeys.235.113267

**Published:** 2023-11-15

**Authors:** Zhen-Hua Zhu, Ngoc Bon Trinh, Thanh Son Hoang, Bo Li

**Affiliations:** 1 College of Agronomy, Jiangxi Agricultural University, Nanchang 330045, China Jiangxi Agricultural University Nanchang China; 2 Silviculture Research Institute, Vietnamese Academy of Forest Sciences, Hanoi 10000, Vietnam Silviculture Research Institute, Vietnamese Academy of Forest Sciences Hanoi Vietnam; 3 Center for Integrative Conservation Biology, Xishuangbanna Tropical Botanical Garden, Chinese Academy of Sciences, Mengla 666303, Yunnan, China Center for Integrative Conservation Biology, Xishuangbanna Tropical Botanical Garden, Chinese Academy of Sciences Mengla China

**Keywords:** *Camptotheca*, lectotype, Northern Vietnam, Nyssaceae

## Abstract

As a primary source of anticancer camptothecin, *Camptotheca* (Nyssaceae) is an economically valuable genus and has long been recorded as endemic to China. Here, *Camptotheca* is reported as a new record to the flora of Vietnam with the discovery of a wild population of *C.acuminata* from Lai Chau Province of northern Vietnam. Based on the consultation of relevant literature and type specimens of *C.acuminata*, a lectotype of the species is designated. Photographic illustrations, morphological description and a distribution map of *C.acuminata* is provided, and a key to all known species of Nyssaceae in Vietnam is presented, too.

## ﻿Introduction

Nyssaceae is a small angiosperm family phylogenetically placed in the order Cornales (Angiosperm Phylogeny Group 2016). It has been reduced to be a subfamily (namely Nyssoideae) of Cornaceae (Angiosperm Phylogeny Group 2009; Reveal and Chase 2011) or divided into three separated smaller families (Mastixiaceae, Davidiaceae, and Nyssaceae) (Thomas et al. 2021). Within Cornales, the phylogenetic position of Nyssaceae has been controversial. It was supported to be a sister of either Curtisiaceae in the analysis of nuclear genomes (Zhang et al. 2020), a clade comprised of Grubbiaceae and Curtisiaceae using an Angiosperms353 dataset (Thomas et al. 2021), or another clade formed by Hydrostachyaceae, Hydrangeaceae, and Loasaceae in chloroplast phylogenies (Schenk and Hufford 2010; Fu et al. 2019; Li et al. 2021). As currently circumscribed, five genera are recognized in Nyssaceae, viz., *Mastixia* Blume, *Davidia* Baill., *Nyssa* L., *Diplopanax* Hand.-Mazz., and *Camptotheca* Decne (Stevens 2001 onwards).

Within Nyssaceae, *Camptotheca* is a distinct genus and could be readily distinguished from other genera by its samaralike fruits clustered in a globose head (Qin and Chamlong 2007). In previous molecular phylogenetic analyses, a sister relationship between *Camptotheca* and *Nyssa* was solidly supported no matter using nuclear or plastid data (Xiang et al. 2011; Chen et al. 2016; Fu et al. 2017, 2019; Li et al. 2019, 2021; Thomas et al. 2021). *Camptotheca* has long been recorded as an endemic genus of seed plants in China (Fang et al. 1983; Ying and Zhang 1994; Qin and Chamlong 2007). Since the publication of the type species, *C.acuminata* Decne., four additional taxa have been described in the genus, viz., C.acuminatavar.tenuifolia W.P. Fang & Soong, C.acuminatavar.rotundifolia B.M. Yang & L.D. Duan, *C.yunnanensis* Dode, and *C.lowreyana* S.Y. Li. However, the first three names have been treated as synonyms of *C.acuminata*. As currently recognized in Flora of China (Qin and Chamlong 2007), the two species, *C.acuminata* and *C.lowreyana*, can be distinguished by the shapes and number of lateral veins of leaves (Li 1997; Qin and Chamlong 2007).

*Camptotheca* is an ecologically and economically important genus, which do not only play a great role in landscaping (Zhang et al. 2004; Yang et al. 2012; Du 2013) but also is one of the most valuable woody medicine resources (Feng et al. 2000; Li et al. 2002). Ever since a special alkaloid, camptothecin, was successfully isolated from *C.acuminata* (Wall 1966), many studies have focused on its powerful anticancer effects (Venditto and Simanek 2010; Martino et al. 2017; Wang et al. 2023), and related drugs have been developed and approved for treating various cancers (Khaiwa et al. 2021; Jiao et al. 2023). Because of its great value and potential uses, *C.acuminata* was included in the List of National Key Protected Wild Plants of China (National Forestry Administration 1999).

**Figure 1.**
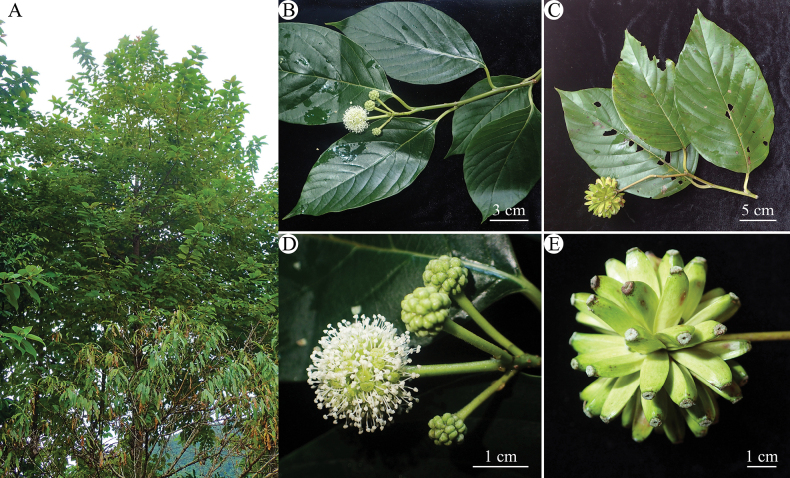
*Camptothecaacuminata***A** habitat and habit **B** flowering branch **C** fruiting branch **D** inflorescences **E** fruits.

In 2022, we encountered a small population of unknown trees without flowers and fruits in Phong Tho District of Lai Chau Province, northern Vietnam. When revisiting the locality from May to July 2023, we successfully collected flowering and fruiting specimens of this tree. After the consultation of relevant literature (Li 1997; Qin and Chamlong 2007) and comparison of type as well as other herbarium specimens, we confidently confirmed its identity as *C.acuminata* based on its morphology (Fig. 1), which appears to be the first record of the species and the genus *Camptotheca* for the flora of Vietnam.

## ﻿Materials and methods

Field surveys were carried out in northern Vietnam from 2022 and 2023. Voucher specimens of *Camptothecaacuminata* were collected from Phong Tho District, Lai Chau Province, and deposited in the herbarium of the Vietnamese Academy of Forest Sciences (**VAFS**).

High-resolution images of the type materials of *C.acuminata* held at the Muséum National d’Histoire Naturelle (P, acronyms according to Thiers 2023+) and the herbarium of Royal Botanic Gardens, Kew (K) were examined via the JSTOR Global Plants website (https://plants.jstor.org/). Other herbarium specimens (mainly from CAF, GXMI, HITBC, IBK, IBSC, KUN, NAS, and PE) were consulted via the Chinese Virtual Herbarium platform (https://www.cvh.ac.cn/index.php). The distribution map is prepared using data obtained from herbaria records as well as our field observations.

## ﻿Taxonomic treatment

### ﻿ Camptotheca acuminata

Taxon classificationPlantaeCornalesNyssaceae

Decaisne Bull. Soc. Bot. France. 20: 157 (1873).

76D7818A-B80D-5F67-A294-DB7C8E11E124

#### Lectotype.

(designated here):—China. Kiang-si (Jiangxi Province): Vallée du Ly-Chan (Lushan Mountain), 1868, *A.David 866* (lectotype P00545522! [image available at http://coldb.mnhn.fr/catalognumber/mnhn/p/p00545522]; isolectotypes: K000704811! [image available at https://plants.jstor.org/stable/10.5555/al.ap.specimen.k000704811] P00545523! [image available at http://coldb.mnhn.fr/catalognumber/mnhn/p/p00545523], P00545524! [image available at http://coldb.mnhn.fr/catalognumber/mnhn/p/p00545524], P00545525! [image available at http://coldb.mnhn.fr/catalognumber/mnhn/p/p00545525]).

#### Description.

Trees deciduous, to 20 m high; bark light gray, deeply furrowed; young branchlets cylindrical, purplish, with gray pubescence, villous; old branchlets glabrous, sparse round or oval lenticels. Leaves alternative; petiole 1.5–3 cm, flat or slightly grooved above, round below, puberulent when young, and almost glabrous mature, blackish when dry; leaf blade papery, 12–28 × 6–12 cm, oblong-ovate, oblong-elliptic or orbicular, base subrounded, margin entire, apex acute, slightly pubescent and pale green adaxially, greenish and lucid abaxially; pinnate veins both surfaces sparsely pubescent, midrib prominent both surfaces, lateral veins (4–)8–11(–15) pairs, slightly prominent adaxially or slightly prominent only near base abaxially. Inflorescence head, terminal or axillary, 1.5–2 in diam.; peduncle 4–6 cm, cylindrical, puberulent when young, then glabrous. Flowers polygamous; bracts 3, triangular, 2.5–3 mm, both surfaces pubescent; calyx cup-shaped, shallowly 5 lobed; lobes toothed; petals 5, caducous, light green, ca. 2 mm; disk conspicuous; stamens 10, outer 5 longer than, glabrous; filaments slender; anthers tetradymous; style ca. 4 mm, glabrous; stigmas 2. Fruit thinly winged, clustered in a globose head, green when young, yellowish brown after drying, 2.5–3.5 cm × 5–7 mm. Seed 1. Cotyledons lanceolate, 2–4× ca. 1 cm, pinniveined, with 6–8 lateral veins on each side.

#### Illustrations.

Fang and Su (1981: 316, fig. 120: 1–7); Yu (1993: 277, fig. 4: 351); Li (1997: 351–352, fig. 1–2); Wu et al. (2007: 322, fig. 322: 1–3).

#### Phenology.

Flower: May-July, fruit: September.

#### Distribution and habitat.

**Figure 2.**
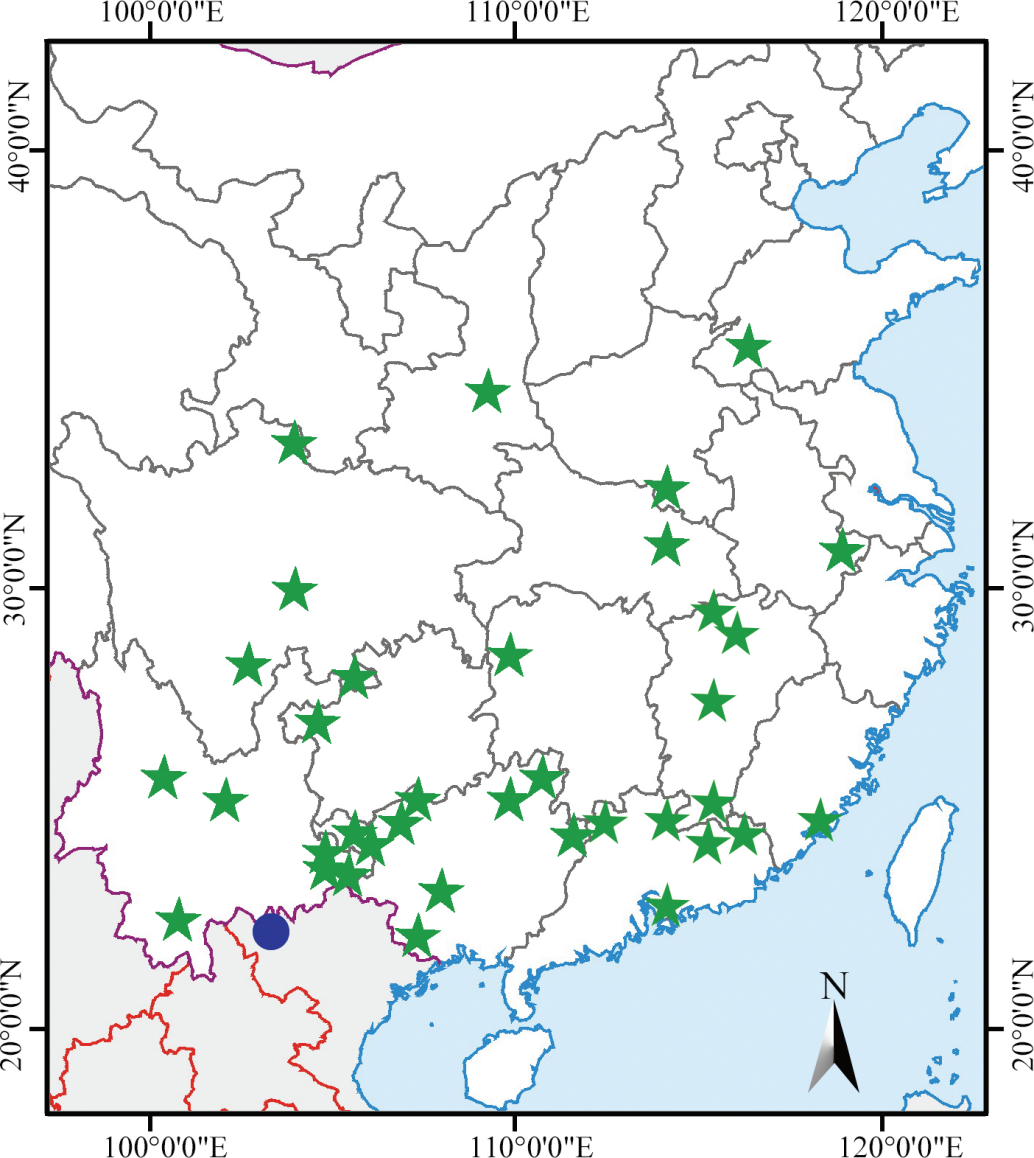
Distribution of *Camptothecaacuminata* in China (green stars) and Vietnam (blue circle).

*Camptothecaacuminata* is widely distributed in southern China provinces (Fujian, Guangdong, Guangxi, Guizhou, Hubei, Hunan, Jiangsu, Jiangxi, Sichuan, Yunnan, Zhejiang), and always grows near riverbanks and forest margins below alt. 1000 m. The newly discovered population of *C.acuminata* is located in northern Vietnam and near the China-Vietnam borders (Fig. 2).

#### Additional specimens examined.

China, Fujian Province: Xiamen City, Siming District, Wanshi Botanical Garden, roadside, elev. 56 m, 24°27'20.2"N, 118°5'38.1"E, 24 June 2020, fr. *T.Wang 402* (AU!); Hubei Province: Xiaogan City, Dawu County, elev. 445 m, 31°43'5.8"N, 114°19'8.9"E, 03 Nov. 2020, fr. *C.Dai DC78* (HIB!); Sichuan Province: Leshan City, E’bian Yi Autonomous County, roadsides under forests, elev. 961 m, 29°18'53"N, 103°16'56"E, 25 Nov. 2017, fr. *X.J.Li LiXJ830* (KUN!); Anhui Province: Xuancheng City, Jingde County, Hui River Protection Area, riversides, elev. 169 m, 30°22'37.2"N, 118°22'55.2"E, 18 Oct. 2016, fr. *W.Zhang and H.F.Wang ANUB02040* (ANUB!); Guizhou Province: Bijie City, Zhijin County, Qimo Town, Sanjiashan Village, broadleaf forests, elev. 1324 m, 26°41'55.2"N, 105°46'58.4"E, 07 Aug. 2015, fr. *L.Chen 522425150807009LY* (GZTM!); Jiangxi Province: Ganzhou City, Xinfeng County, Jinpen Mountain, valley, elev. 375 m, 25°13'32"N, 115°12'44"E, 03 Oct. 2014, fr. *R.P.Kuang LXP03-04734* (HNNU!); Hunan Province: Xiangxi Autonomous Prefecture, Baojing County, Fuxing Town, elev. 397 m, 28°38'53.9"N, 109°44'58.6"E, 12 Aug. 2012, fr. *X.J.Su and H.B.Liu 433125D00090811017* (JIU!). Vietnam. Lai Chau Province: Phong Tho District, Pa Ve Su commune, Mu Sang, Vang Ma Chai, in forests, elev. 1150 m, 22°39'38.48"N, 103°15'29.56"E, 11 June 2023, fr. *T.S.Hoang 22039* (VAFS).

#### Note.

In the protologue of *Camptothecaacuminata*, Decaisne (1873) noted its type locality as “Thibet orientale, prov. Moupin, Ly-chan valley” which was proved to be erroneous according to the examination of David’s original collection labels (Franchet 1884). In fact, the type gathering (*A.David 866*) was collected by Father Armand David in 1868 from Lushan Mountain of Jiangxi Province, eastern China (Franchet 1884). When tracing the gathering, we sorted out four separate specimens held at the Muséum National d’Histoire Naturelle (P) and one deposited in the herbarium of Royal Botanic Gardens, Kew (K), and confirmed that not a single specimen has been designated as the type. Thus, we here propose the best preserved one simultaneously having flowers and fruits (barcode no. P00545522) as the lectotype of *C.acuminata* in accordance with the Article 9.3 of the International Code of Nomenclature for algae, fungi, and plants (Shenzhen Code) (Turland et al. 2018).

In Vietnam, only one genus of Nyssaceae was previously recorded, i.e., *Nyssa* L. As currently known, five species of the genus have been discovered in the country (Fang et al. 1983; Qin and Chamlong 2007; Tan and Deng 2016; Tagane et al. 2020), viz., *Nyssajavanica* (Blume) Wangerin, *Nyssasinensis* Oliv., *Nyssabifida* Craib, *Nyssabidoupensis* Tagane & Yahara and *Nyssahongiaoensis* Tagane & Komada. With the supplement of *Camptotheca* and *C.acuminata* to the Vietnamese flora, the Nyssaceae is now represented by two genera and six species. Thus, a key to all species of Nyssaceae in Vietnam is provided below.

### ﻿Key to the genera and species of Nyssaceae in Vietnam

**Table:** 

1	Fruit thinly winged, clustered in a globose head	***Camptotheca* (*C.acuminata*)**
–	Fruit drupaceous, laterally flattened, solitary or several in a cluster	**2 (*Nyssa*)**
2	Trees evergreen	***N.hongiaoensis***
–	Trees deciduous	**3**
3	Flowers pedicellate, in umbels or racemes	***N.sinensis***
–	Flowers sessile or male ones shortly pedicellate, in capitates	4
4	Branchlets glabrous	***N.bidoupensis***
–	Branchlets densely tomentose	**5**
5	Branches and leaves glabrescent to subglabrous when mature	***N.javanica***
–	Branches and leaves persistently densely tomentose	***N.bifida***

## Supplementary Material

XML Treatment for Camptotheca acuminata

## ﻿Additional information

### Author ORCIDs

Zhen-Hua Zhu https://orcid.org/0000-0003-1351-0942

Ngoc Bon Trinh https://orcid.org/0000-0001-7530-8008

Thanh Son Hoang https://orcid.org/0000-0003-0351-929X

Bo Li https://orcid.org/0000-0003-1628-8128

### Data availability

All of the data that support the findings of this study are available in the main text.
